# Glycoprotein IIb/IIIa Inhibitors May Modulate the Clinical Benefit of Radial Access as Compared to Femoral Access in Primary Percutaneous Coronary Intervention: A Meta-Regression and Meta-Analysis of Randomized Trials

**DOI:** 10.1155/2021/9917407

**Published:** 2021-06-15

**Authors:** Stefano Rigattieri, Ernesto Cristiano, Francesca Giovannelli, Antonella Tommasino, Francesco Cava, Barbara Citoni, Domenico Maria Zardi, Andrea Berni, Massimo Volpe

**Affiliations:** ^1^Interventional Cardiology Unit, Sant'Andrea University Hospital, Rome, Italy; ^2^Department of Clinical and Molecular Medicine, School of Medicine and Psychology, Sapienza University of Rome, 00189 Rome, Italy

## Abstract

**Objectives:**

Several randomized controlled trials (RCTs) consistently reported better clinical outcomes with radial as compared to femoral access for primary percutaneous coronary intervention (PCI). Nevertheless, heterogeneous use of potent antiplatelet drugs, such as Gp IIb/IIIa inhibitors (GPI), across different studies could have biased the results in favor of radial access. We performed an updated meta-analysis and meta-regression of RCTs in order to appraise whether the use of GPI had an impact on pooled estimates of clinical outcomes according to vascular access.

**Methods:**

We computed pooled estimates by the random-effects model for the following outcomes: mortality, major adverse cardiovascular events (death, myocardial infarction, stroke, and target vessel revascularization), and major bleedings. Additionally, we performed meta-regression analysis to investigate the impact of GPI use on pooled estimates of clinical outcomes.

**Results:**

We analyzed 14 randomized controlled trials and 11090 patients who were treated by radial (5497) and femoral access (5593), respectively. Radial access was associated with better outcomes for mortality (risk difference 0.01 (0.00, 0.01), *p*=0.03), MACE (risk difference 0.01 (0.00, 0.02), *p*=0.003), and major bleedings (risk difference 0.01 (0.00, 0.02), *p*=0.02). At meta-regression, we observed a significant correlation of mortality with both GPI use (*p*=0.011) and year of publication (*p*=0.0073), whereas no correlation was observed with major bleedings.

**Conclusions:**

In this meta-analysis, the use of radial access for primary PCI was associated with better clinical outcomes as compared to femoral access. However, the effect size on mortality was modulated by GPI rate, with greater benefit of radial access in studies with larger use of these drugs.

## 1. Introduction

Several trials and meta-analyses consistently showed better clinical outcomes with radial as compared to femoral access for primary PCI in patients with ST-elevation myocardial infarction (STEMI), mainly because of a striking reduction of bleeding events related to vascular access site [1, 2]. Bleedings negatively impact prognosis in acute coronary syndromes [3]; therefore, several bleeding avoidance strategies, including radial access, femoral vascular closure devices (VCD), and safer antithrombotic drugs, such as bivalirudin, have been adopted in order to improve outcomes [4]. Consequently, the use of potent antiplatelet agents known to increase hemorrhagic risk, such as Gp IIb/IIIa inhibitors (GPI) [5], has declined in recent years [6] and is now mostly recommended for bail-out by clinical practice guidelines [7]. This change in practice is reflected in randomized trials comparing radial and femoral access, with variable reported rate of GPI use (generally higher in previous trials and lower in contemporary trials). We performed an updated meta-analysis and meta-regression of randomized trials aiming to investigate whether the rate of GPI use may affect the extent of benefit of radial as compared to femoral access for primary PCI.

## 2. Materials and Methods

We performed a study-level meta-analysis of randomized trials comparing radial to femoral access and including patients with STEMI undergoing primary PCI. Major electronic databases (PubMed, Scopus, and the Cochrane Library) were searched from inception through December 2020 using the following terms: “(trans)radial,” “(trans)femoral,” “primary percutaneous coronary intervention,” “ST-elevation myocardial infarction,” and “randomized controlled trial.” We limited our search to articles published in English language on peer-reviewed Journals; the “Similar articles” section of PubMed and references from selected studies were also checked. The following clinical end-points were considered for analysis: (1) in-hospital or 30-day mortality for all causes (according to study definition), (2) major bleedings, and (3) major adverse cardiovascular events (MACE). Major bleedings were defined according to the TIMI criteria or, alternatively, according to the definition provided by each study. Procedural success rate was also appraised. Two investigators independently performed the literature search, screened studies for eligibility, and extracted data using a standardized collection form (SR and EC). Disagreement was resolved by consensus. For studies comparing radial and femoral access in the whole spectrum of acute coronary syndromes, we only considered outcomes relative to the STEMI subgroup. This analysis was planned in accordance with the Preferred Reporting Items for Systematic Reviews and Meta-Analysis protocols [8]. Data were extracted onto standard spreadsheets, based on a standardized data configuration protocol. The Cochrane Collaboration tool was used to assess the risk of bias in randomized controlled trials [9]. The study quality was also evaluated according to a score, expressed on an ordinal scale, allocating 1 point for the presence of each of the following: (1) statement of objectives, (2) explicit inclusion and exclusion criteria, (3) description of interventions, (4) objective means of follow-up, (5) description of adverse events, (6) power analysis, (7) description of statistical methods, (8) multicenter design, (9) discussion of withdrawals, and (10) details on medical therapy (e.g., antithrombotic regimens) during and after coronary procedures [10]. For dichotomous variables, pooled statistics were calculated as weighted risk differences (RD) with 95% confidence intervals (CIs) using the random-effects DerSimonian and Laird model [11, 12]. The number needed to treat (NNT) was calculated according to the following formula: NNT = 1/RD. We tested heterogeneity of the included studies with Q statistics and the extent of inconsistency between results with *I* [2] statistics, which describe the percentage of total variation across studies that is due to heterogeneity. Heterogeneity is described as low, moderate, and high, based on *I* [2] values of 25%, 50%, and 75%, respectively. Presence of publication bias was visually estimated by constructing funnel plots. Sensitivity analyses were performed using the fixed effects model and a leave-one-out analysis to assess whether the pooled results were influenced by a single trial. To assess whether the proportion of patients receiving GPI modulates study-specific estimates (RDs of mortality, major bleeding, and MACE between radial and femoral access), a random-effects restricted maximum likelihood meta-regression analysis was conducted [13]. Statistical significance was set at *p* < 0.05 (2-tailed). Statistical analyses were carried out using the Review Manager 5.3 software (available from http://tech.cochrane.org/revman) and the Comprehensive Meta-Analysis 3.0 software (Biostat, Englewood, NJ, USA).

## 3. Results

**Figure 1:**
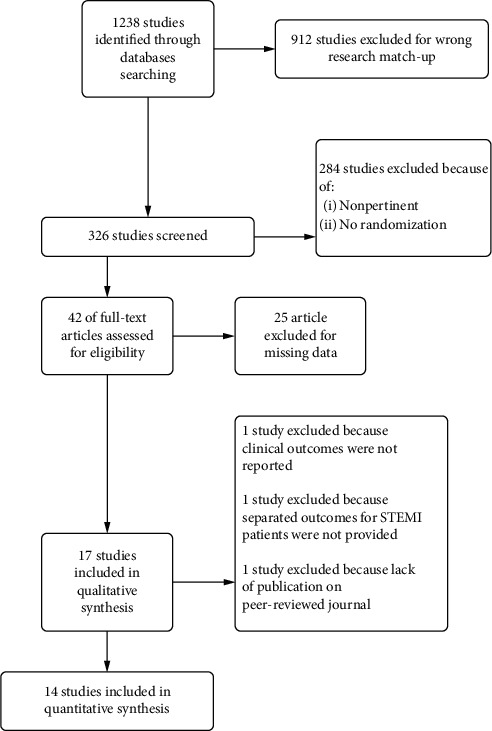
Preferred Reporting Items for Systematic Reviews and Meta-Analysis flow diagram.

**Figure 2:**
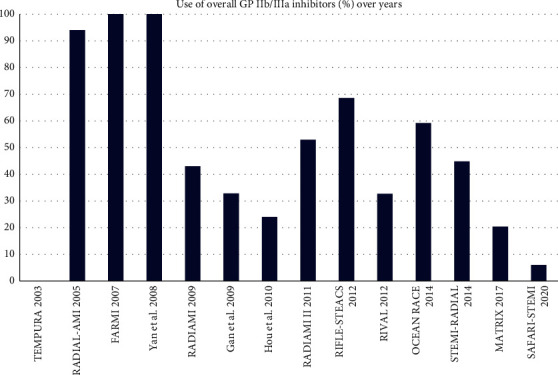
Overall prevalence of glycoprotein IIb/IIIa inhibitors use in the included studies.

**Figure 3:**
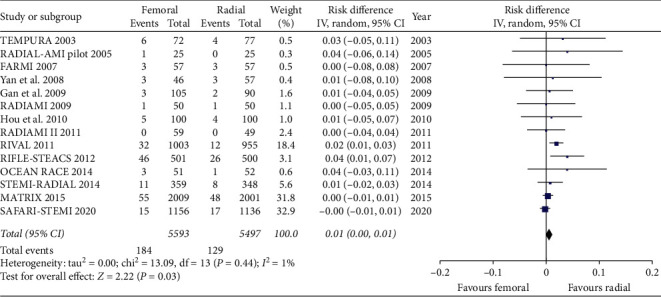
Forest plot showing all-cause mortality between radial and femoral access.

**Figure 4:**
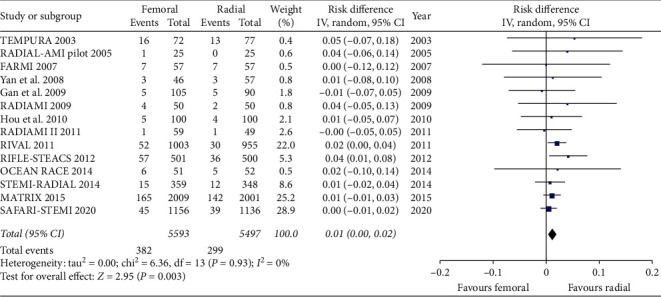
Forest plot showing major adverse cardiovascular events between radial and femoral access.

**Figure 5:**
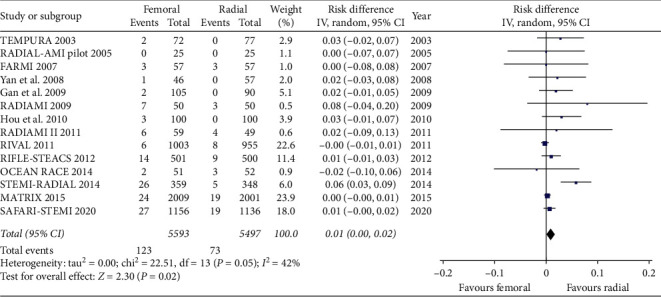
Forest plot showing TIMI major bleedings between radial and femoral access.

**Figure 6:**
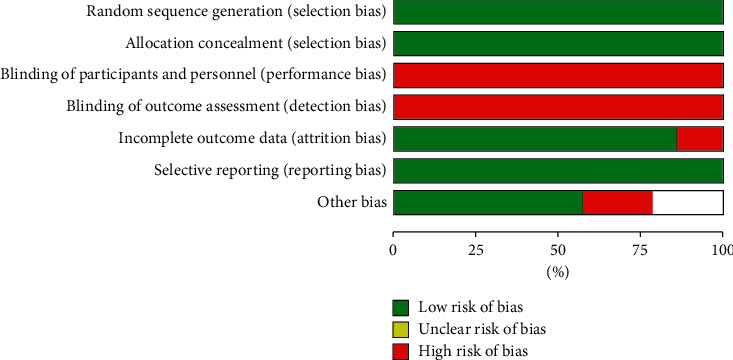
Cochrane Collaboration risk of bias graph.

**Figure 7:**
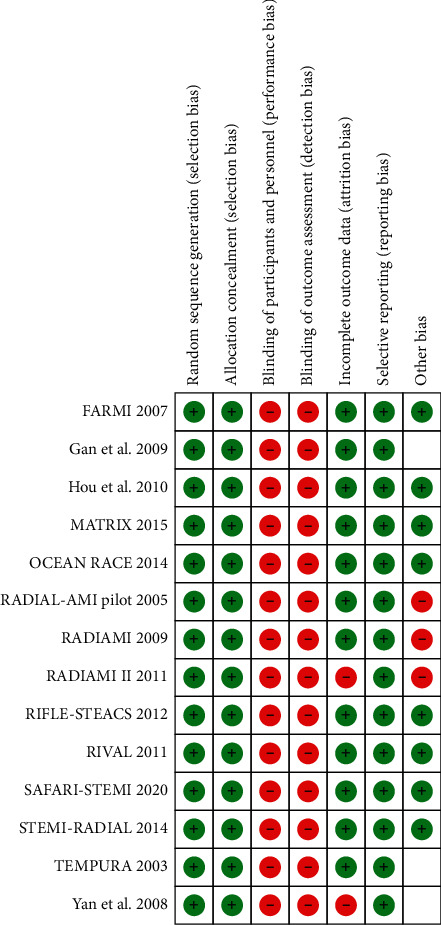
Cochrane Collaboration risk of bias summary.

**Figure 8:**
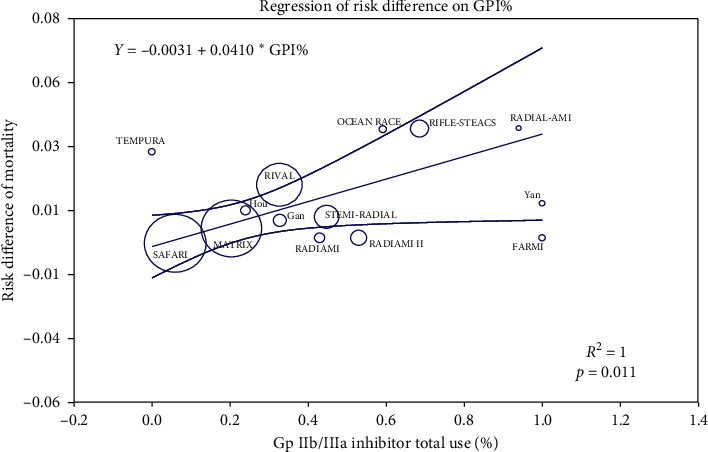
Meta-regression graph describing the effect of the prevalence of glycoprotein IIb/IIIa inhibitors use on the risk difference for mortality according to vascular access (radial or femoral).

We included in this meta-analysis 14 randomized trials enrolling a total of 11090 patients randomly allocated to radial (*n* = 5497) or femoral access (*n* = 5593) [14–27]. The different steps of the search through the Preferred Reporting Items for Systematic Reviews and Meta-Analysis flow diagram are illustrated in Figure 1, whereas the summary of included trials is reported in Table 1. The overall rate of GPI ranged from 0% to 100%, with an average of 48.5% (Figure 2). Data about the use of femoral VCD were inconsistently reported across studies; no data were reported by 6 studies; whereas in the remaining, the rate of VCD use ranged from 0% to 93% (average 25.9%). Cardiogenic shock was an exclusion criterion in most studies. Pooled rates of mortality were 3.29% in femoral arm and 2.35% in radial arm (risk difference 0.01 (0.00, 0.01), *p*=0.03, NNT 167) with a very low heterogeneity across studies (*I*^2^ = 1%, Figure 3). Pooled rates of MACE were 6.83% in femoral arm and 5.44% in radial arm (risk difference 0.01 (0.00, 0.02), *p*=0.003, NNT 83) with no heterogeneity across studies (*I*^2^ = 0%, Figure 4). Pooled rates of major bleedings were 2.19% in femoral arm and 1.33% in radial arm (risk difference 0.01 (0.00, 0.02), *p*=0.02, NNT 100) with moderate heterogeneity across studies (*I*^2^ = 42%, Figure 5). Procedural success rate was similar in femoral and radial arm (88.07% vs. 88.41%, *p*=0.54). The Cochrane Collaboration risk of bias graph and summary are reported in Figures 6 and 7, respectively; all studies presented a performance bias (no blinding of participants and personnel) and a detection bias (no blinding of outcome assessment). Similar results were obtained using the fixed effects model and leave-one-out analysis. Visual inspection of funnel plots showed asymmetry for the outcome “major bleedings.” Meta-regression indicated a significant correlation with mortality for both rate of GPI use (*r*^2^ = 100%, coefficient 0.0410, 95% CI 0.0092–0.0728, *p*=0.011; Figure 8) and year of publication (*r*^2^ = 100%, coefficient −0.0021, 95% CI −0.0036 to −0.0006, *p*=0.0073), whereas no significant correlation was observed between major bleedings and rate of GPI use (*p*=0.65).

## 4. Discussion

The main results of this meta-analysis can be summarized as follows: (1) in patients with STEMI undergoing primary PCI, radial access is associated to a significantly reduced risk of all-cause mortality, major bleeding, and MACE as compared to femoral access; (2) GPI may act as modulators of the effect size on mortality, with higher benefit of radial as compared to femoral access in studies with higher rate of use of these drugs; (3) there is a significant interaction between years of publication and effect size on mortality, with higher benefit of radial as compared to femoral access in earlier studies.

Radial access for PCI was introduced in the early 1990s and gained popularity being associated to increased patient comfort and less access site complications as compared to femoral access [28, 29]. In the following years, the detrimental prognostic impact of bleedings was acknowledged, especially in acute coronary syndrome patients [3, 30]; therefore, radial access was increasingly recognized as an effective way to reduce access-related bleedings. Several randomized trials and meta-analyses, conducted in a 15-years frame, consistently showed the superiority of radial over femoral access for PCI on several clinical end-points, including mortality [31]. Differently, in SAFARI-STEMI, the last randomized trials published so far, no significant differences were observed between the 2 sites of access both in 30-day all-cause mortality and in major bleedings, although the trial was prematurely terminated for futility [27]. Several factors have been taken into account in order to explain these findings; among these, the most relevant are the high rate of the use of bleeding avoidance strategies in SAFARI-STEMI, including bivalirudin and VCD in the femoral arm, and the low, single-digit rate of Gp IIb/IIIa administration. The latter point prompted us to investigate, through meta-regression, whether the effect size of vascular access site on clinical outcomes could be modulated by different use of potent antiplatelet drugs. Indeed, we observed a strong correlation between rate of GPI use and benefit of radial access on the risk of all-cause mortality, in contrast with a recently published study, although study inclusion criteria and, therefore, included studies were different [32]. Interestingly, we did not observe a significant correlation between rate of GPI use and major bleedings. Although this finding is counterintuitive and is in contrast with the proposed mechanism of benefit of radial access (less bleedings translating in less mortality), another recently published, comprehensive meta-analysis of studies comparing radial and femoral access for coronary angiography and PCI reported a lack of correlation between major bleeding and mortality estimates [33]. In our opinion, there are 3 possible explanation for these findings: (1) potent antithrombotic agents, such as GPI, administered in patients with STEMI markedly increase the risk of nonaccess site bleeding, possibly diluting the treatment effect on bleeding according to access site; (2) different from mortality, myocardial infarction, or stroke, the adjudication of bleeding events in clinical trial is more difficult, and there is marked heterogeneity in reporting this outcome across different studies, although we tried to reduce such variability by adopting the TIMI classification when provided by the authors; (3) apart from GPI use, other factors may play a role in modulating the effect size associated with the selection of vascular access. In this regard, the correlation between years of publication and effect size on mortality that we observed in the present study is particularly interesting in the light of a recently reported correlation between years of publication and major bleedings, with greater benefit with radial access in studies published before 2010 [33]. Indeed, one could argue that the advantage of radial over femoral access on both mortality and bleeding events may have been mitigated through years not only by a progressive decline in the use of GPI but also by other factors, including refinements in operator skills and device technology and a growing expertise in the management of vascular access and closure. Notwithstanding the lack of firm evidence [34], the use of femoral vascular closure device was associated with a mortality benefit in a propensity-matched analysis of a large database [35].

Our study presents several limitations. First of all, this is a study-level, not a patient-level meta-analysis, providing average treatment effects; this needs to be taken into account when exploring associations between clinical outcomes and average rates of use of GPIs, as we did in the present study. Second, data about femoral vascular closure device use were inconsistently reported among the included studies. Third, as previously outlined, the definition of major bleeding varied widely among the included studies.

In conclusion, our meta-analysis confirms previous findings showing the superiority of radial as compared to femoral access in the reduction of mortality, major bleedings, and MACE in STEMI patients undergoing primary PCI. Moreover, our study suggests that the benefit of radial access may be modulated by different rates of GPI use and, possibly, by the implementation of other bleeding avoidance strategies, such as femoral VCD, although their impact could not be formally assessed.

## Figures and Tables

**Table 1:** Characteristics of randomized controlled trials included in the meta-analysis.

Study, year	Femoral arm, *n* (%)	Radial arm, *n* (%)	Mean age (SD)	Male gender (%)	Follow-up length	GP IIb/IIIa in femoral arm, *n* (%)	Gp IIb\IIIa in radial arm, *n* (%)	Overall Gp IIb/IIIa use, *n* (%)	Femoral VCD, *n* (%)	Major bleeding definition	Study quality
TEMPURA, 2003	72 (48.3)	77 (51.7)	66 (11)	121 (81.2)	In hospital	0 (0.0)	0 (0.0)	0.0	NR	TIMI	9
RADIAL-AMI, 2005	25 (50.0)	25 (50.0)	55 (12)	44 (88)	30 days	23 (92.0)	24 (96.0)	94.0	2 (8.0)	Intracranial or intraperitoneal bleeding, a drop in hemoglobin ≥ 5 g/dl or hematocrit ≥ 15% or whole blood or packed red cell transfusions	8
FARMI, 2007	57 (50.0)	57 (50)	59 (12)	96 (84.2)	In hospital	57 (100.0)	57 (100.0)	100.0	0 (0)	TIMI	8
Yan et al., 2008	46 (44.7)	57 (55.3)	70.8 (8)	77 (74.8)	30 days	46 (100.0)	57 (100.0)	100.0	0 (0)	TIMI	7
Gan et al., 2009	105 (53.8)	90 (46.2)	52.9 (12)	157 (80.5)	In hospital	36 (34.3)	28 (31.1)	32.8	0 (0)	Not specified	6
RADIAMI, 2009	50 (50.0)	50 (50.0)	59.5 (91.1)	68 (68)	In hospital	21 (42.0)	22 (44.0)	43.0	NR	Fatal bleeding, bleeding requiring blood transfusion, operation, or resulting in >3 gr/dl hemoglobin drop, intracranial hemorrhage	8
Hou et al., 2010	100 (50.0)	100 (50.0)	65.6 (8)	141 (70.5)	30 days	20 (20.0)	28 (28.0)	24.0	0 (0)	Hemoglobin drop ≥ 2 mmol/l, blood transfusion, need for vascular repair	6
RADIAMI II, 2011	59 (54.6)	49 (45.4)	59.6 (10)	69 (64)	In hospital	32 (54)	25 (51.0)	53.0	55 (93)	Bleeding resulting in death or needing transfusion or surgical intervention, hemoglobin drop > 3 gr/dl, intercranial bleeding	8
RIVAL, 2012	1003 (51.2)	955 (48.8)	60 (11)	1548 (79.1)	30 days	312 (31.1)	329 (34.5)	32.7	NR	TIMI and ACUITY	10
RIFLE-STEACS, 2012	501 (50.1)	500 (49.9)	65 (10)	734 (73.4)	30 days	350 (69.9)	337 (67.4)	68.6	NR	TIMI	10
STEMI-RADIAL, 2014	359 (50.8)	348 (49.2)	62.1 (11.5)	546 (77)	30 days	162 (45.1)	155 (45.5)	44.8	136 (38)	HORIZONS-AMI	10
OCEAN RACE, 2014	51 (49.5)	52 (50.5)	62 (11)	79 (77)	In hospital	30 (58.8)	31 (59.6)	59.2	NR	REPLACE-2	8
MATRIX, 2017	2009 (50.1)	2001 (49.9)	63.9 (12)	3093 (77.1)	30 days	383 (19.1)	435 (21.7)	20.4	NR	BARC, TIMI, and GUSTO	10
SAFARI-STEMI, 2020	1156 (50.4)	1136 (49.6)	62 (12)	1784 (77.8)	30 days	68 (5.9)	69 (6.1)	6.0	789 (68)	TIMI and BARC	10

## Data Availability

The data used to support this META-ANALYSIS are from previously reported studies and datasets, which have been cited. The processed data are available from the corresponding author upon request.

## References

[1] Osman M., Saleem M., Osman K. (2020). Radial versus femoral access for percutaneous coronary intervention in patients with ST-segment elevation myocardial infarction: trial sequential analysis. *American Heart Journal*.

[2] Ferrante G., Rao S. V., Jüni P. (2016). Radial versus femoral access for coronary interventions across the entire spectrum of patients with coronary artery disease. *JACC: Cardiovascular Interventions*.

[3] Valgimigli M., Costa F., Lokhnygina Y. (2017). Trade-off of myocardial infarction vs. bleeding types on mortality after acute coronary syndrome: lessons from the Thrombin Receptor Antagonist for Clinical Event Reduction in Acute Coronary Syndrome (TRACER) randomized trial. *European Heart Journal*.

[4] Singh M. (2015). Bleeding avoidance strategies during percutaneous coronary interventions. *Journal of the American College of Cardiology*.

[5] Safley D. M., Venkitachalam L., Kennedy K. F., Cohen D. J. (2015). Impact of glycoprotein IIb/IIIa inhibition in contemporary percutaneous coronary intervention for acute coronary syndromes. *JACC: Cardiovascular Interventions*.

[6] Gellatly R. M., Connell C., Tan C. (2020). Trends of use and outcomes associated with glycoprotein-IIb/IIIa inhibitors in patients with acute coronary syndromes undergoing percutaneous coronary intervention. *Annals of Pharmacotherapy*.

[7] Neumann F. J., Sousa-Uva M., Ahlsson A. (2019). 2018 ESC/EACTS Guidelines on myocardial revascularization. *European Heart Journal*.

[8] Shamseer L., Moher D., Clarke M. (2015). Preferred reporting items for systematic review and meta-analysis protocols (prisma-p) 2015: elaboration and explanation. *BMJ*.

[9] Higgins J. P. T., Altman D. G., Gotzsche P. C. (2011). The Cochrane Collaboration’s tool for assessing risk of bias in randomised trials. *BMJ*.

[10] Jadad A. R., Moore R. A., Carroll D. (1996). Assessing the quality of reports of randomized clinical trials: is blinding necessary?. *Controlled Clinical Trials*.

[11] Walter S. D. (2000). Choice of effect measure for epidemiological data. *Journal of Clinical Epidemiology*.

[12] DerSimonian R., Laird N. (1986). Meta-analysis in clinical trials. *Controlled Clinical Trials*.

[13] Thompson S. G., Higgins J. P. T. (2002). How should meta-regression analyses be undertaken and interpreted?. *Statistics in Medicine*.

[14] Saito S., Tanaka S., Hiroe Y. (2003). Comparative study on transradial approach vs. transfemoral approach in primary stent implantation for patients with acute myocardial infarction: results of the Test for Myocardial infarction by Prospective Unicenter Randomization for Access sites (TEMPURA) trial. *Catheterization and Cardiovascular Interventions*.

[15] Cantor W. J., Puley G., Natarajan M. K. (2005). Radial versus femoral access for emergent percutaneous coronary intervention with adjunct glycoprotein IIb/IIIa inhibition in acute myocardial infarction-the RADIAL-AMI pilot randomized trial. *American Heart Journal*.

[16] Brasselet C., Tassan S., Nazeyrollas P., Hamon M., Metz D. (2007). Randomised comparison of femoral versus radial approach for percutaneous coronary intervention using abciximab in acute myocardial infarction: results of the FARMI Trial. *Heart*.

[17] Yan Z. X., Zhou Y. J., Zhao Y. X (2008). Safety and feasibility of transradial approach for primary percutaneous coronary intervention in elderly patients with acute myocardial infarction. *Chinese Medical Journal*.

[18] Gan L., Lib Q., Liuc R., Zhaoc Y., Qiuc J., Liao Y. (2009). Effectiveness and feasibility of transradial approaches for primary percutaneous coronary intervention in patients with acute myocardial infarction. *Journal of Nanjing Medical University*.

[19] Chodór P., Krupa H., Kurek T (2009). RADIal versus femoral approach for percutaneous coronary interventions in patients with acute myocardial infarction (RADIAMI): a prospective, randomized, single-center clinical trial. *Cardiology Journal*.

[20] Chodór P., Kurek T., Kowalczuk A (2011). Radial vs. femoral approach with StarClose clip placement for primary percutaneous coronary intervention in patients with ST-elevation myocardial infarction. RADIAMI II: a prospective, randomised, single centre trial. *Kardiologia Polska*.

[21] Hou L., Wei Y. D., Li W. M., Xu Y. W. (2010). Comparative study on transradial versus transfemoral approach for primary percutaneous coronary intervention in Chinese patients with acute myocardial infarction. *Saudi Medical Journal*.

[22] Jolly S. S., Yusuf S., Cairns J. (2011). Radial versus femoral access for coronary angiography and intervention in patients with acute coronary syndromes (RIVAL): a randomised, parallel group, multicentre trial. *The Lancet*.

[23] Romagnoli E., Biondi-Zoccai G., Sciahbasi A. (2012). Radial versus femoral randomized investigation in ST-segment elevation acute coronary syndrome. *Journal of the American College of Cardiology*.

[24] Bernat I., Horak D., Stasek J. (2014). ST-segment elevation myocardial infarction treated by radial or femoral approach in a multicenter randomized clinical trial. *Journal of the American College of Cardiology*.

[25] Kołtowski Ł., Filipiak K. J., Kochman J. (2014). Access for percutaneous coronary intervention in ST segment elevation myocardial infarction: radial vs. femoral—a prospective, randomised clinical trial (OCEAN RACE). *Kardiologia Polska*.

[26] Vranckx P., Frigoli E., Rothenbühler M. (2017). Radial versus femoral access in patients with acute coronary syndromes with or without ST-segment elevation. *European Heart Journal*.

[27] Le May M., Wells G., So D. (2020). Safety and efficacy of femoral access vs. radial access in ST-segment elevation myocardial infarction. *JAMA Cardiology*.

[28] Kiemeneij F., Laarman G. J., Odekerken D., Slagboom T., Van Der Wieken R. (1997). A randomized comparison of percutaneous transluminal coronary angioplasty by the radial, brachial and femoral approaches: the access study. *Journal of the American College of Cardiology*.

[29] Agostoni P., Biondi-Zoccai G. G. L., De Benedictis M. L. (2004). Radial versus femoral approach for percutaneous coronary diagnostic and interventional procedures. *Journal of the American College of Cardiology*.

[30] Segev A., Strauss B. H., Tan M., Constance C., Langer A., Goodman S. G. (2005). Predictors and 1-year outcome of major bleeding in patients with non-ST-elevation acute coronary syndromes: insights from the Canadian Acute Coronary Syndrome Registries. *American Heart Journal*.

[31] Karrowni W., Vyas A., Giacomino B. (2013). Radial versus femoral access for primary percutaneous interventions in ST-segment elevation myocardial infarction patients. *JACC: Cardiovascular Interventions*.

[32] Jhand A., Atti V., Gwon Y. (2021). Meta-analysis of transradial vs. transfemoral access for percutaneous coronary intervention in patients with ST elevation myocardial infarction. *The American Journal of Cardiology*.

[33] Chiarito M., Cao D., Nicolas J. (2021). Radial versus femoral access for coronary interventions: an updated systematic review and meta‐analysis of randomized trials. *Catheterization and Cardiovascular Interventions*.

[34] Biancari F., D’Andrea V., Marco C. D., Savino G., Tiozzo V., Catania A. (2010). Meta-analysis of randomized trials on the efficacy of vascular closure devices after diagnostic angiography and angioplasty. *American Heart Journal*.

[35] Farooq V., Goedhart D., Ludman P., De Belder M. A., Harcombe A., El-Omar M. (2016). Relationship between femoral vascular closure devices and short-term mortality from 271 845 percutaneous coronary intervention procedures performed in the United Kingdom between 2006 and 2011. *Circulation: Cardiovascular Interventions*.

